# Inadequate consumption of vitamin A-rich foods among preschool children in Wolaita Sodo, Southern Ethiopia

**DOI:** 10.3389/fnut.2024.1503040

**Published:** 2024-12-24

**Authors:** Selamawit Mathewos Mekisso, Samson Kastro Dake, Dibora Teferi Haile, Debritu Nane

**Affiliations:** ^1^Department of Reproductive Health and Nutrition, School of Public Health, College of Health Science and Medicine, Wolaita Sodo University, Wolaita Sodo, Ethiopia; ^2^Health and Nutrition Camp Supervisor for the Nguenyyiel Refugee, Action Against Hunger, Gambela, Ethiopia

**Keywords:** vitamin A-rich food, animal source food, preschool children, Ethiopia, inadequate

## Abstract

**Background:**

The primary cause of vitamin A deficiency in developing countries like Ethiopia is the inadequate consumption of vitamin A-rich foods. Preschool children are particularly vulnerable due to their higher nutritional requirements and increased susceptibility to infections. This study aims to assess the prevalence of inadequate consumption of vitamin A-rich foods and identify the associated factors among preschool children in Wolaita Sodo, Southern Ethiopia.

**Methods:**

A community-based cross-sectional study was conducted using multi-stage sampling to select 471 households with preschool children between July 15 and August 15, 2021. Data analysis was performed using SPSS version 25. Binary logistic regression was employed to identify predictors of inadequate consumption of vitamin A-rich foods. Variables with a *p*-value <0.25 in the bivariate analysis were included in the multivariable logistic regression. The strength of the associations was estimated using adjusted odds ratios with 95% confidence intervals. Statistical significance was determined at a *p*-value <0.05.

**Result:**

The prevalence of inadequate consumption of Vitamin A-rich foods among pre-school children in this study was 381 (81.1%) with a 95% confidence interval of 77.3 to 84.9%. Predictors for inadequate consumption of Vitamin A rich foods were being a girl [AOR = 0.41, 95% CI: 0.24, 0.69], aged 46–59 months [AOR = 0.46, 95% CI: 0.23, 0.93], rural residence [AOR = 2.36, 95% CI: 1.22, 4.57], family size of five or more [AOR = 2.36, 95% CI: 1.15, 4.86], household income of <2000 Ethiopian Birr [AOR = 3.98, 95% CI: 1.18, 13.40], and morbidity in last 2 weeks [AOR = 0.36, 95% CI: 0.17, 0.74].

**Conclusion:**

This study showed that the participants’ consumption of vitamin A-rich food was inadequate. Greater emphasis be placed on food-based tactics to increase pre-schoolers’ intake of foods high in vitamin A. Enhancing socioeconomic status is also crucial for increasing the intake of foods high in vitamin A.

## Background

Pro-vitamin A carotenoid, or preformed vitamin A, is a fat-soluble vitamin that is naturally present in diet (1). Vitamin A plays a crucial role in maintaining the integrity of epithelial cells, supporting growth, immunity, vision, and various metabolic processes (2, 3). Since the human body cannot synthesize vitamin A, it must be obtained from dietary sources (2). The best sources of preformed vitamin A are foods derived from animals, such as red meat, poultry, fish liver, animal liver, milk, whole eggs, butter, cheese, cream, fatty fish, and foods fortified with vitamin A. Plant sources rich in pro-vitamin A include dark green leafy vegetables, carrots, pumpkins, sweet potatoes, yams, watermelon, and yellow or orange fruits like ripe papaya, mango, and squash (4, 5).

Vitamin A deficiency (VAD) is multi-causal but main cause is inadequate dietary intake (6), and other causes are impaired absorption of vitamin A and/or demand increased due to infections (7). VAD is characterized by a weakened immune system, stunted growth, xerophthalmia, night blindness, and blindness (8). Common illnesses in developing countries like Measles, diarrhea, malaria, and other infectious illness morbidity and death in children are linked to VAD (9). Globally, VAD is a significant public health issue, with Africa and Southeast Asia bearing the brunt of the disease (10). Because of their greater need for the nutrients necessary for their quick growth and development as well as their higher incidence of various diseases, preschool-aged children are the most vulnerable population in the community (10).

To prevent VAD, the World Health Organization (WHO) recommends a combination of strategies, including dietary diversification, food fortification, and vitamin A supplementation (11, 12). In regions where VAD is a public health concern, children aged 6 to 59 months receive vitamin A supplements twice a year (12). However, supplementation alone is not sufficient to address the root causes of VAD. A diet rich in vitamin A-rich foods is essential for achieving a sustained and effective increase in serum retinol levels, thereby preventing VAD (13, 14).

In many low-income countries, the diets of most people lack sufficient nutrients to support healthy child development (15). Several studies conducted in these regions have shown that preschool-aged children often do not consume enough vitamin A-rich foods (16–18).

Studies on dietary habits in various population groups across Ethiopia have also shown that preschool children in different regions consume insufficient amounts of vitamin A-rich foods (19–21). However, there is limited information regarding the consumption of vitamin A-rich foods among Ethiopian preschoolers. Therefore, this study aimed to assess the prevalence of inadequate consumption of vitamin A-rich food and identify the associated factors among preschool children in Wolaita Sodo, Southern Ethiopia.

## Methods

### Study setting

Wolaita Sodo town is the administrative capital for Wolaita zonal administration which is found in Southern Ethiopia. The town is located 380 km south of Addis Ababa. There are 25 kebeles (lower administrative units) in the town. The total population of the town is estimated to be 244,817 in 2021; 25,537 preschool school. Potato, sweet potato, cassava, yam, banana, *enset* (Enset ventricosum), maize, haricot bean, *teff* (*Eragrostis tef* (Zucc.)), Sorghum, Pumpkin, broad Bean, Peas, Kidney Bean and Chick-pea are main food crops being produced in the surrounding area. One public and four private hospitals, three health centers, and more than 20 private clinics deliver health services to the population in the town and the surrounding areas.

### Study design and period

A community-based cross-sectional study involving preschool children paired with their primary caretakers was conducted in Wolaita Sodo town from July to August 2021.

### Population and sampling

The source population for this study consisted of all preschool children paired with their primary caretakers in Wolaita Sodo town. The study population included a sample of selected preschool children and their primary caretakers. The sample size was calculated to be 520 using a double population proportion formula, based on the following assumptions: a 95% confidence level, a 5% margin of error, an estimated prevalence of vitamin A-rich food consumption of 28.8% taken from a similar study in Ethiopia (3), a design effect of 1.5, and a 5% non-response rate. A multistage sampling technique was used to select the study participants. First, the *kebeles* in the town were stratified into semi urban and rural by assuming socio-economic differences. Among the 25 *kebeles* (14 semi urban and 11 urban) in the town, five semi urban and three urban *kebeles* were selected randomly to make the sampling representative. The total sample size was then proportionally allocated to each selected *kebele* based on their respective populations. For the second stage of sampling, households with preschool children were chosen using the Simple Random Sampling method until the allocated sample size for each *kebele* was reached In households with more than one preschool child, one child was randomly selected using a lottery method to ensure equal representation. If primary caretaker-child pairs were not available during the initial visit, two follow-up visits were made. If the pair remained unavailable after these attempts, they were excluded from the study to maintain data collection timelines.

### Data collection

A structured interviewer-administered questionnaire was adopted from relevant articles and related literature (22). Local qualitative market surveys and focus group discussions with mothers were conducted to identify and document vitamin A-rich foods available in the area. The Helen Keller international (HKI) qualitative food frequency questionnaire (FFQ) was adopted to the local context (23). A 7-days FFQ through modified Helen Keller international FFQ was used to assess the consumption of vitamin A-rich foods and a 24-h dietary diversity questionnaire was used to assess the dietary diversity. The questionnaire was pre-tested on 5% of the respondents in areas not included in the actual study and no adjustments were made after the pre-testing. Four data collectors and two supervisors who have previous experience in similar studies were recruited and trained for 2 days on the modules of the questionnaire, selection of study participants, and ethics.

### Variables

#### Outcome variable

The consumption of vitamin A-rich foods was the outcome variable, categorized as adequate if participants consumed vitamin A-rich animal source foods for at least 4 days per week, or consumed a combination of vitamin A-rich animal and plant source foods for at least 6 days per week (23).

#### Exposure variables and covariates

##### Socio-demographic and socio-economic

Age of the child, sex of the child, marital status of parents, Education and occupation status of parents, and monthly income of the household.

##### Maternal and child health

Maternal antenatal care (ANC) follow-up for the index child, child morbidity, breastfeeding practice for the index child.

##### Dietary diversity score

Dietary diversity score (DDS) was used to determine the dietary diversity. A single 24-h recall of childrens’ consumption of commonly consumed foods was used to collect information for DDS (24). We have categorized the foods into 10 groups based on Food and Agriculture Organization (FAO) recommendations; (1) starch stable food, (2) vegetables, (3) fruits, (4) meat, (5) egg, (6) fish and other seafood, (7) legumes, nuts and seeds, (8) milk and milk products, (9) oil and fats, (10) sweets, spices, condiments and beverage (25). The response categories were “Yes” if at least one food item in the group was consumed and “No” when a food item in the group was not consumed. We have classified the results into <4 food items (poor DDS) and ≥ 4 food items (good DDS) (26).

### Statistical analysis

Data were entered into Epi-Data version 4.6 and analysed by using SPSS version 25. Statistics such as frequency, percentage, mean, and standard deviation of the mean were performed for the main variables. Binary regression analysis was used to select exposure variables with an association to the outcome. Exposure variables with *a p*-value of less than 0.25 in the bivariate analysis were considered candidate for a multivariate regression analysis to control for possible confounders. The fitness of the multivariate regression model was tested by using Hosmer-Lemeshow goodness of fit and it was considered a fitted model (*p*-value of 0.31). Multivariate regression analysis was used to identify independent predictors for vitamin A-rich foods consumption. A *p*-value <0.05 was considered for statistical significance and adjusted odds ratio (AOR) with 95% CIs were reported.

## Results

### Socio-demographic characteristics

The overall response rate of this study was 90.6%. The dropout was primarily due to the availability issues of the subjects. More than half (56.9%) of the respondents live in urban areas, while 43.1% are from rural areas. There is a slightly higher proportion of boys (56.5%) compared to girls (43.5%) among the preschool children surveyed. One fourth (25.1%) of studied children were in the age group of 36–45 months. The majority (92.4%) of the respondents were mothers of the children. Concerning respondents’ age, most respondents (45.2%) are in the age group of 20–29 years, closely followed by those aged 30 and older (47.6%). Regarding marital status, almost all respondents (97.0%) were currently married. Regarding religion, the highest percentage of respondents identified as Protestant (50.3%). In terms of education, the largest group (25.9%) completed primary education, while 40.3% of respondents were housewives. The household size showed that the majority (75.4%) had five or more family members, and 56.5% of households had one child under five. Finally, half of the respondents (50.3%) reported a monthly income between 2,000–4,000 Ethiopian Birr (ETB) (Table 1).

**Table 1:** Socio-demographic and socio-economic characteristics of the study participants, Wolaita Sodo, Ethiopia.

Variable (*n* = 472)	Category	Frequency	Percentage
Place of residence	Urban	268	56.9
	Rural	203	43.1
Sex of the child	Boys	266	56.5
	Girls	205	43.5
Age of the child (in months)	24–35	175	37.2
	36–45	118	25.1
	46–59	178	37.8
Respondents relation to the child	Mother	435	92.4
	Not the mother	36	7.6
Age of the respondents	<20	34	7.2
	20–29	213	45.2
	30+	224	47.6
Marital status of the respondents	Currently married	457	97.0
	Currently not married	14	2.9
Religion of the respondents	Protestant	237	50.3
	Orthodox	171	36.3
	Muslim	29	6.2
	Catholic	34	7.2
Educational status of the respondents	No formal education	51	10.8
	Primary (1–6)	122	25.9
	Lower secondary (7–10)	98	20.8
	Higher secondary (11–12)	110	23.4
	Above secondary	90	19.1
Occupation of the respondents	Housewife	190	40.3
	Merchant	169	35.9
	Gov’t employee	87	18.5
	Other^a^	25	5.3
Household family size	<5	116	24.6
	5+	355	75.4
Number of under five children	1	266	56.5
	2+	205	43.5
Household monthly income (ETB)	<2000	66	14.0
	2000–4,000	237	50.3
	>4,000	168	35.7

a Daily laborers and students.

This table provides an overview of the sociodemographic characteristics of the study participants. It includes details about the participants’ place of residence, sex and age of the children, relationship to the child, marital status, religious affiliation, educational background, and occupation. Additionally, the table describes household characteristics such as family size, the number of children under five, and household income levels.

### Maternal and child health and nutrition related characteristics

In the present study, (91.9%) of the mothers attended ANC follow-up for the index child. Almost all the mothers, (98.7%) gave birth to the index child at the health facility. More than half, (55.2%) of the children were being breastfed during the study period, and (58.6%) started complementary feeding exactly at 6 months of age. About (13.2%) of the children had history of morbidity in the last 2 weeks preceding the study (Table 2).

**Table 2:** Maternal and child health and nutrition related characteristics of the study participants, Wolaita Sodo, Ethiopia.

Variable (n = 472)	Category	Frequency	Percentage
Attended ANC follow up	Yes	433	91.9
	No	38	8.1
Place of delivery	Health facility	465	98.7
	Home	6	1.3
Currently breastfeeding	Yes	260	55.2
	No	211	44.8
Time of complementary feeding initiation	Before 6 months	6	1.3
	At 6 months	276	58.6
	After 6 months	189	40.1
Morbidity in the last 2 weeks	Yes	62	13.2
	No	409	86.8

This table summarizes key maternal and child health characteristics among study participants. It includes information on antenatal care (ANC) attendance, place of delivery, current breastfeeding status, timing of complementary feeding initiation, and recent morbidity in children.

### Vitamin A-rich foods consumption and dietary diversity score

In the current study, the majority, (93.6%) did not consume adequate meat, poultry or fish and one fourth (75.2%) did not consume adequate egg. The majority of the children, (83.2%) were found to have good dietary diversity score (see Table 3).

**Table 3:** Vitamin A-rich food consumption and dietary diversity score of the study participants, Wolaita Sodo, Ethiopia.

Variable (*n* = 472)	Category	Frequency	Percentage
Meat, poultry and fish consumption	Adequate	30	6.4
	Inadequate	441	93.6
Egg consumption	Adequate	117	24.8
	Inadequate	354	75.2
Vitamin A-rich plant sources consumption	Adequate	169	35.9
	Inadequate	302	64.1
Dietary diversity score	Poor (<4)	79	16.8
	Good (≥ 4)	392	83.2

This table outlines the dietary patterns and diversity among preschool children in the study. It includes data on the adequacy of meat, poultry, fish, egg, and vitamin A-rich plant source consumption. Additionally, it highlights the dietary diversity score, categorized as either poor or good.

### Prevalence of inadequate consumption of vitamin A-rich foods

**Figure 1:**
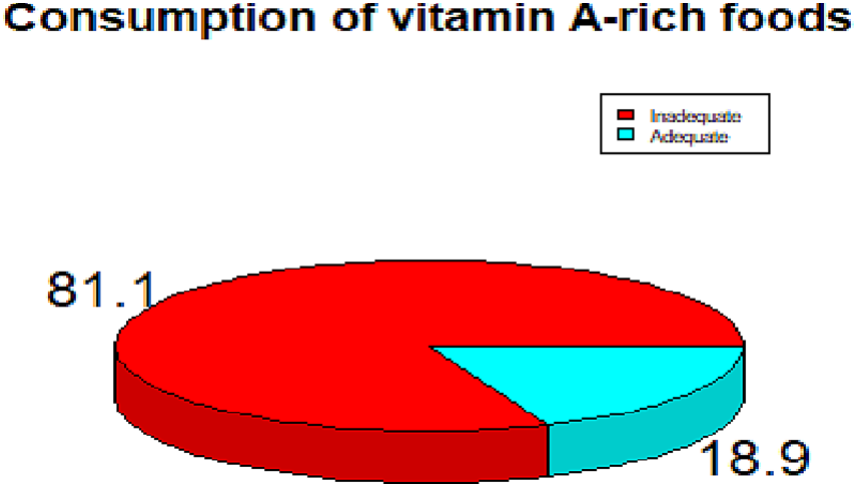
Consumption of vitamin A-rich foods among the study participants.

The prevalence of inadequate consumption of vitamin A rich foods in the current study was 381(81.0%) [95% CI: 77.3, 84.9] (Figure 1).

### Factors associated with inadequate consumption of vitamin A-rich foods

In the multivariate logistic regression, residence, sex of the child, age of the child, family size, and previous history of illness were found to be significantly associated with inadequate consumption of vitamin A rich foods among preschool children. Pre-school children who reside in rural areas were about two times more likely to have inadequate vitamin A-rich food consumption compared to their counterparts [AOR = 2.36, 95% CI: 1.22, 4.57]. Girls had 59% lower chance of having inadequate vitamin A-rich food consumption compared to their counterparts [AOR = 0.41, 95% CI: 0.24, 0.69]. Pre-school children aged 46–59 month were 54% less likely to have inadequate vitamin A-rich food consumption compared to children aged 24–35 months [AOR = 0.46, 95% CI: 0.23, 0.93]. Pre-school children who reside in households with a family size of five or more were about two times more likely to have inadequate vitamin A-rich food consumption [AOR = 2.36, 95% CI: 1.15, 4.86] compared to those who reside in households with less than 5 members. Children from households with income <2000 ETB were about four times more likely to have inadequate vitamin A-rich food consumption compared to those who have income >4,000 ETB [AOR = 3.98, 95% CI: 1.18, 13.40]. Pre-school children who had morbidity in the last 2 weeks prior to the data collection were 64% less likely to have inadequate vitamin A-rich food consumption compared to their counterparts [AOR = 0.36, 95% CI: 0.17, 0.74] (Table 4).

**Table 4:** Factors associated with vitamin A-rich food consumption, Wolaita Sodo, Ethiopia.

Variables (*n* = 471)	Vitamin A consumption		COR (95% CI)	AOR (95% CI)
	Adequate	Inadequate		
Residence				
Urban	70 (78.7)	198 (51.8)	1	1
Rural	19 (21.3)	184(48.2)	3.42[1.99, 5.91]	2.36[1.22, 4.57]**
Sex of the child				
Boy	35 (39.3)	231(60.5)	1	1
Girl	54 (60.7)	151(39.5)	0.42[0.26, 0.68]	0.41[0.24, 0.69]***
Age of child (months)				
24–35	26 (29.2)	149 (39.0)	1	1
36–45	21(23.6)	97 (25.4)	0.81[0.43, 1.51]	0.64[0.30, 1.37]
46–59	42 (47.2)	136 (35.6)	0.57[0.33, 0.97]	0.46[0.23, 0.93]**
Caregiver’s relation to the child				
Mother	78 (87.6)	357 (93.5)	2.01[0.95, 4.26]	1.39[0.37, 5.32]
Other	11 (12.4)	25 (6.5)	1	1
No of under 5 children				
1	40 (44.9)	226 (59.2)	1.78[1.12, 2.83]	0.75[0.40, 1.41]
2+	49 (55.1)	156 (40.8)	1	1
Caregivers’ education				
No formal education	5 (5.6)	46 (12.0)	3.16[1.12, 8.91]	1.39[0.37, 5.32]
Primary	20 (22.5)	102 (26.7)	1.75[0.89, 3.44]	1.44[0.48, 4.32]
Lower secondary	12 (13.5)	86 (22.5)	2.46[1.14, 5.30]	2.16[0.72, 6.50]
Higher secondary	29 (32.6)	81 (21.2)	0.96[0.51, 1.81]	0.86[0.32, 2.27]
Above secondary	23 (25.8)	67 (17.5)	1	1
Caregivers’ occupation				
Housewife	27 (30.3)	163 (42.7)	1	1
Merchant	39 (43.8)	130 (34.0)	0.55[0.32, 0.95]	0.56[0.29, 1.07]
Gov’t employee	19 (21.3)	68 (17.8)	0.59[0.31, 1.14]	0.89[0.32, 2.49]
Others^a^	4 (4.5)	21 (5.5)	0.87[0.28, 2.73]	0.77[0.21, 2.92]
Family size				
<5	73 (82.0)	282 (73.8)	1	1
5+	16 (18.0)	100 (26.2)	1.62[0.90, 2.91]	2.36[1.15, 4.86]**
Household income				
<2000	4 (4.5)	62 (16.2)	5.50[1.89, 16.00]	3.98[1.18, 13.40]**
2000–4,000	41(46.1)	196 (51.3)	1.70[1.05, 2.75]	1.33[0.74, 2.37]
>4,000	44 (49.4)	124 (32.5)	1	1
Attended ANC follow up				
Yes	15 (16.9)	23 (6.0)	1	
No	74 (83.1)	359 (94.0)	3.16[1.58, 6.35]	2.17[0.96, 4.93]
Currently breast feeding the child				
Yes	44 (49.4)	216 (56.5)	1	1
No	45 (50.6)	166 (43.5)	0.75[0.47, 1.19]	0.76[0.44, 1.29]
Had morbidity				
Yes	20 (22.5)	42 (11.0)	2.35[1.30, 4.24]	0.36[0.17, 0.74]***
No	69 (77.5)	340 (89.0)	1	1

a Daily labourer, student.

** *p*-value less than 0.05; *** *p*-value less than 0.01.

This table presents the logistic regression analysis of sociodemographic factors associated with adequate vitamin A consumption among children. Variables include place of residence, child’s sex and age, caregiver’s relation, education, occupation, family size, income, ANC attendance, breastfeeding, and morbidity history. The table reports crude and adjusted odds ratios (COR, AOR) with 95% confidence intervals, identifying significant factors influencing vitamin A consumption.

## Discussion

Consumption of foods rich in vitamin A is low in developing countries. In this study, preschool children in Wolaita Sodo, Southern Ethiopia were assessed for their consumption of vitamin A-rich foods and associated characteristics. Three hundred eighty one (81.1%) of the preschool children reported inadequate consumption of vitamin A-rich foods. In studies conducted in other parts of Ethiopia, inadequate consumption of vitamin A-rich foods from animal and plant sources was 98 and 84.4%, respectively (19, 21). In the current study, the prevalence of inadequate consumption of vitamin A-rich foods was less as compared to the above. The possible reason for this could be the difference in study area. The previous studies were conducted in rural setting whereas the current study was conducted in urban setting.

On the other hand, a study conducted in East Africa reported a comparable prevalence (80.8%) of inadequate consumption of vitamin A-rich foods in Burundi and a much lower prevalence (34.1%) in Kenya (27). The difference in results can be explained by the effect of sample size, differences in the study population, tools used to measure the outcome variables across the studies, and study settings differences. According to WHO recommendation, preschool children should take vitamin A-rich foods every day to prevent VAD (28), and also HKI method says inadequacy should not exceed 70% of the community (29). Therefore, the study finding is still an alarming proportion according to the recommendations.

From the studied preschool children, 302 (64.1%) did not consume vitamin A-rich plant foods 24 h preceding the study time. In addition, the proportion of inadequate consumption of animal sources of vitamin-A, meat, poultry, or fish was 441(93.6%), chicken egg 354 (75.2%) and milk/milk product was 205 (43.5%). This low consumption of animal sources of vitamin A is in agreement with other studies in Burkina Faso (18), South Africa (30), and Addis Ababa in Ethiopia (31). This informs us that much of vitamin A sources comes from plant source foods. It has been reported that in developing countries almost 80% of vitamin A source foods are plant sources (32).

Place of residence, sex of the child, age of the child, family size, level of income, and history of morbidity were found to be significantly associated with inadequate consumption of vitamin A-rich foods among preschool children. Pre-school children who reside in rural areas were about two times more likely to have inadequate vitamin A-rich food consumption compared to their counterparts. This finding is in line with the study conducted in Bangladesh (33). This might be because rural resident caretakers were less educated and have lack of awareness to feed vitamin A-rich foods.

Girls were about 59% less likely to have inadequate Vitamin A-rich food consumption compared to boys. This finding is different from other studies conducted in India (34), Poland (35), and Kenya (36) where girls were at increased risk for lower consumption of vitamin A-rich foods. In the contrary, a study done in Rural Mexico reported that there was no significant gender difference in food consumption (37). Similar to our study findings, another study conducted in England reported that the intake of vitamin A was higher among girls, though the difference is minor and statistically insignificant (38). There have been no convincing physiologically based studies that could account for the intrinsic factor of sex difference. This suggests that cultural factors could more likely explain the difference.

Pre-school children aged 46–59 months were 54% less likely to have inadequate vitamin A-rich food consumption compared to those aged 24–35 months. This finding is supported by a study conducted in Southern Ethiopia which indicated that an increase in child’s age was significantly associated with a decreased likelihood of not consuming vitamin A-rich foods (16). This could be because caretakers know that they have to increase the amount of vitamin A-rich food as children grow up.

Pre-school children who reside in households with a family size of greater than or equal to five were about two times more likely to have inadequate vitamin A-rich food consumption. This is in line with a study conducted in Northern Ethiopia (39). This could be because large family size reduces food consumption.

Children from households with income <2000 ETB were about four times more likely to have inadequate vitamin A-rich food consumption compared to those whose income is >4,000 ETB. Studies conducted in India, Mexico, and Kenya using different dietary approaches has also shown that consumption of micronutrient-rich foods, especially animal foods, is strongly correlated with household affluence (40). This could be because animal source foods are more expensive than plant source foods for households will lower income to purchase.

Pre-school children who had no morbidity in the last 2 weeks before the data collection were 64% less likely to have inadequate vitamin A-rich food consumption compared to their counterparts. This report was reinforced by a study finding from rural Kenya where food intake reduction was greater in children with illnesses like gastrointestinal illness, lower respiratory tract infection, measles, and other febrile illnesses (41). This is in line with other study findings from different parts of Ethiopia (42, 43). This was because since illness and low dietary intake are interrelated with one another, the illness will lead preschool children to loss of appetite which will result in poor consumption.

This study is a cross-sectional study and thus it is difficult to identify cause effect relationship. We also did not consider seasonal variation in food consumption which might affect food consumption pattern. In addition, information on the amount of consumption of vitamin A-rich foods was not collected. Furthermore, the study did not measure vitamin A deficiency directly, as it did not include assessments of vitamin A status.

## Conclusion

This study revealed that the consumption of vitamin A-rich foods by preschool children was inadequate and they were at risk of developing vitamin A deficiency in this study area. Sex, place of residence, age, history of morbidity of the child, income and family size were the identified predictors for inadequate consumption of vitamin A-rich foods. We recommend giving more attention to food-based strategies to enhance the consumption of vitamin A-rich foods among preschool children. Improving Socio economic status is also very important to improve the consumption of vitamin A-rich foods. In addition, health professionals should encourage preschool children to take more vitamin A-rich foods, particularly when they get sick.

## Data Availability

The raw data supporting the conclusions of this article will be made available by the authors, without undue reservation.
